# Prognostic impact of pretreatment lymphocyte-to-monocyte ratio in advanced epithelial cancers: a meta-analysis

**DOI:** 10.1186/s12935-018-0698-5

**Published:** 2018-12-06

**Authors:** Yiming Mao, Donglai Chen, Shanzhou Duan, Yuhuan Zhao, Changjiang Wu, Feng Zhu, Chang Chen, Yongbing Chen

**Affiliations:** 1 Department of Thoracic Surgery, The Second Affiliated Hospital of Soochow University, 1055 Sanxiang Road, Gusu District, Suzhou, 215004 China; 2 Department of Thoracic Surgery, Shanghai Pulmonary Hospital, Tongji University, School of Medicine, 507 Zhengming Road, Yangpu District, Shanghai, 200433 China; 3 Department of Thoracic Surgery, Suzhou Kowloon Hospital, Shanghai Jiao Tong University School of Medicine, Suzhou, China; 4 Department of Intensive Care Unit, Suzhou Kowloon Hospital, Shanghai Jiao Tong University School of Medicine, Suzhou, China

**Keywords:** Lymphocyte-to-monocyte ratio, Prognosis, Epithelial cancer, Treatment

## Abstract

**Background:**

There is increasing evidence that inflammation-based biomarkers are associated with tumor microenvironment which plays important roles in cancer progression. A high lymphocyte-to-monocyte ratio (LMR), has been suggested to indicate favorable prognoses in various epithelial cancers. We performed a meta-analysis to quantify the prognostic value of LMR in advanced-stage epithelial cancers undergoing various treatment.

**Methods:**

We searched PubMed, EMBASE, Web of science and Cochrane Library up to July 2018 for relevant studies. We included studies assessing the prognostic impact of pretreatment LMR on clinical outcomes in patients with advanced-stage epithelial cancers. The primary outcome was overall survival (OS) and the secondary outcome was progression free survival (PFS). The summary hazard ratio (HR) and 95% confidence interval (CI) were calculated.

**Results:**

A total of 8984 patients from 35 studies were included. A high pretreatment LMR was associated with favorable OS (HR = 0.578, 95% CI 0.522–0.641, *P* < 0.001) and PFS (HR = 0.598, 95% CI 0.465–0.768, *P* < 0.001). The effect of LMR on OS was observed among various tumor types. A higher pretreatment LMR was associated with improved OS in chemotherapy (n = 10, HR = 0.592, 95% CI 0.518–0.676, *P *< 0.001), surgery (n = 10, HR = 0.683, 95% CI 0.579–0.807, *P *< 0.001) and combined therapy (n = 11, HR = 0.507, 95% CI 0.442–0.582, *P *< 0.001) in the subgroup analysis by different therapeutic strategies. The cut-off value for LMR was 3.0 (range = 2.35–5.46). Subgroup analysis according to the cut-off value showed a significant prognostic value of LMR on OS and PFS in both subgroups.

**Conclusions:**

A high pretreatment LMR is associated with favorable clinical outcomes in advanced-stage epithelial cancers undergoing different therapeutic strategies. LMR could be used to improve clinical decision-making regarding treatment in advanced epithelial cancers.

**Electronic supplementary material:**

The online version of this article (10.1186/s12935-018-0698-5) contains supplementary material, which is available to authorized users.

## Background

Cancer remains the most threatening disease to human health worldwide [1]. Although strides in various therapies to treat advanced-stage cancers have never ceased to be made, the long-term survival of cancer patients remains disappointing. Hitherto, the clinical and pathological staging systems have been the primary references used to predict the outcomes of cancer patients; these systems are based on preoperative imaging or biopsy of tumors rather than the individual data [2]. In addition, current staging systems cannot always accurately predict the risk of recurrence and benefits from neoadjuvant or adjuvant therapy in advanced cancers [2–11]. Therefore, more effective and convenient indicators should be taken as supplementary references to stratify cancer patients and to guide therapeutic strategies.

Currently, there is increasing evidence that inflammation-based biomarkers are associated with tumor microenvironment [12–16], which plays important roles in cancer development, progression and metastasis in epithelial cancers. Inflammatory responses in the tumor microenvironment have been reported to be reflected by some common biomarkers in peripheral blood, especially some cytokines, leukocytes and their subtypes [2, 12, 14, 17]. Therefore inflammation-based biomarkers are potential indicators for the prognoses of cancer patients undergoing different treatments.

Numerous studies have reported that the pretreatment LMR is associated with prognosis in various cancers [17–38]. However, the prognostic impact of LMR in advanced epithelial cancers remains inconclusive. The purpose of this meta-analysis is to investigate the association between pretreatment LMR and the outcomes for advanced-stage epithelial cancers with different therapeutic strategies, on the basis of current evidence.

## Methods

### Search strategy

This meta-analysis was conducted in line with the preferred reporting items for systematic reviews and meta-analyses (PRISMA) statement [39]. Studies were identified by searching databases including PubMed, EMBASE, Web of science and Cochrane Library up to June 2018 without language restrictions. The full search strategies are presented in Additional file 1: Table S1. The reference lists of the previously published meta-analyses were also manually reviewed until no additional potential articles could be identified.

### Study selection and inclusion criteria

The identified studies were selected by two independent reviewers (Mao and Chen). First, the titles and abstracts were screened to assess study the eligibility, and then the full text was reviewed. Any disagreement was resolved by discussion or by a third reviewer (Duan) to reach a consensus. Studies meeting that met the following criteria were included: (1) Studies involving individuals with advanced-stage epithelial tumors and concerning the prognostic value of the pretreatment LMR. The definition of “advanced stage” was derived from the original research from which we extracted data. The timing of assessment of LMR was set at baseline before any treatment was initiated. (2) Studies providing the hazard ratio (HR) with a 95% confidence interval (CI) for overall survival (OS) or progression-free survival (PFS), or indirect information such as Kaplan–Meier curves used to estimate survival data on the basis of the methods previously described [2, 40–42]. (3) If the same population was included in two or more studies, only the one study with the largest sample size or the latest information was included. (4) The full text was available. The exclusion criteria were as follows: (1) Non-human research; (2) Case reports, reviews, comments, editorials, letters or conference abstracts; (3) Patients with mesenchymal tumors or hematologic malignancies; (4) Insufficient data for estimating a HR and 95% CI; (5) LMR included only as a continuous variable rather than a dichotomized variable.

### Data extraction and quality assessment

Two reviewers (Mao and Chen) independently carried out the data extraction from the eligible studies. The following information was recorded for each study: first author’s name, year of publication, research region, inclusion period, study design, number of patients, patient age, tumor type, tumor stage, treatment, cut-off value of LMR, time of LMR assessment, follow-up period, study endpoints, analysis of hazard ratios and adjustment variables. The individual HR (with the corresponding 95% CIs) in the studies was also extracted for OS and PFS to assess the therapeutic efficacy. The HRs were preferentially extracted from multivariate analyses. Any discrepancies between reviewers were resolved by consensus. As the previous studies reported [2, 43, 44], a set of modified predefined criteria was applied to assess the risk of bias of the included studies. The modified predefined criteria are shown in Additional file 1: Table S3. Studies with a score of 7 or higher were defined as high-quality, and those with a score whereas scores of less than 7 were considered low-quality.

### Statistical analyses

General data were analyzed using Statistical Package for Social Sciences (SPSS) software (version 21.0 for Windows). STATA 12.0 software (StatCorp, College Station, TX, USA) was used to conduct the meta-analysis. Cochran’s *Q* test and Higgins *I*-squared statistic were used to test the heterogeneity of different studies. A *P* value of less than 0.1 was considered significant. *I*^*2*^> 50% was deemed to show substantial heterogeneity [45]. When the heterogeneity was significant, a random-effect model was applied; otherwise, a fixed-effect model was used. Summary HRs were calculated according to the appropriate model depending on the heterogeneity of the included studies. The reasons for inter-study heterogeneity were explored using subgroup analysis. Sensitivity analysis was also conducted by omission of each single study to evaluate the stability of the results. Publication bias was assessed using funnel plots, Begg’s and Egger’s tests [46, 47]. When publication bias was suggested, Duval and Tweedie trim-and-fill methods were applied for the number of missing studies, and the pooled estimate was recalculated to adjust the primary results [48]. All statistical tests were two-sided, and statistical significance was defined as *P* less than 0.05.

## Results

### Selection of eligible studies

**Fig. 1:**
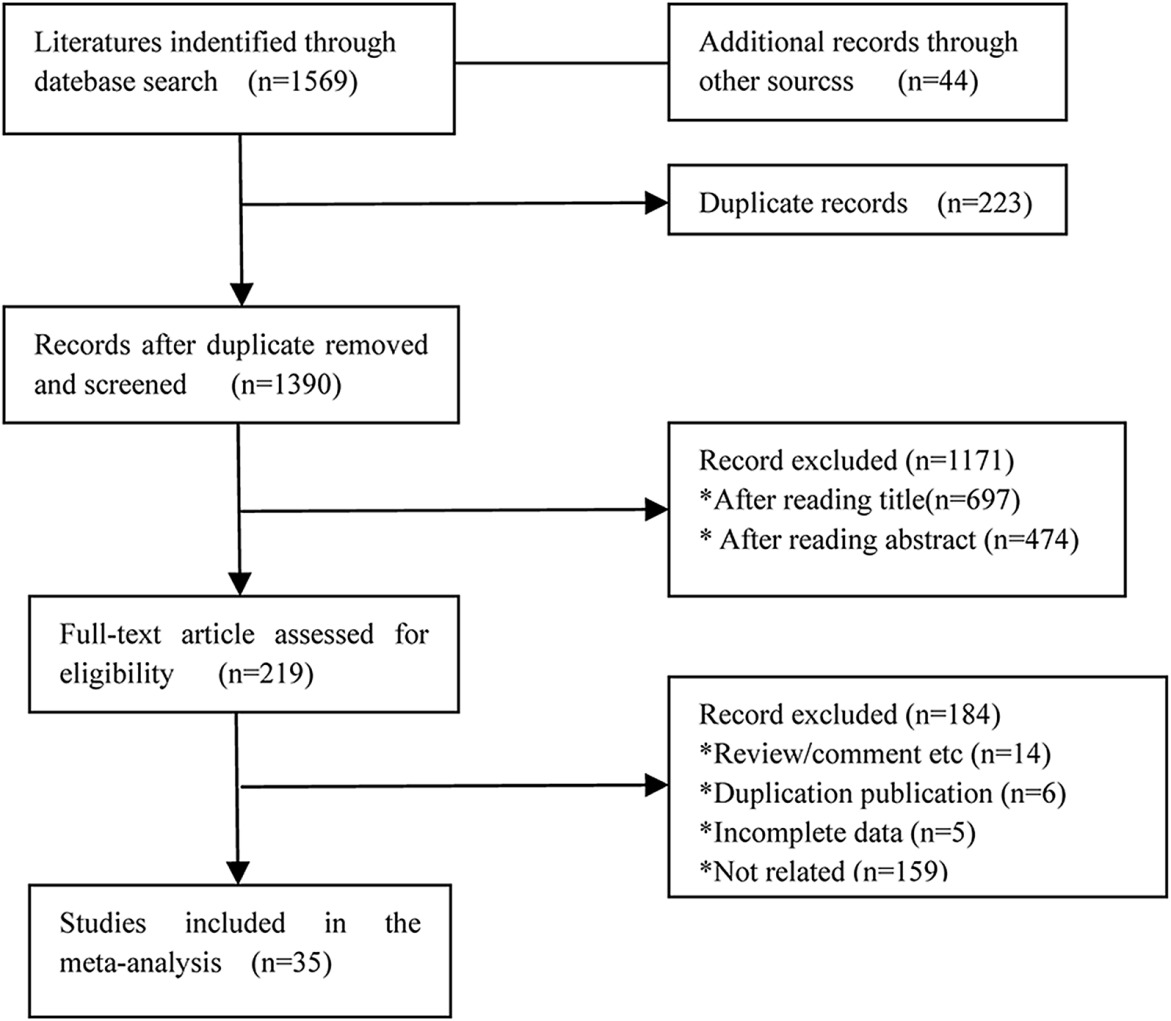
Literature search of eligible studies

The flow chart of the literature search is shown in Fig. 1. In summary, our search strategy identified 1613 studies after searching the relevant online databases. We excluded 223 duplicate records from the initial studies. After screening the title and abstracts of 1390 studies, 1171 studies were removed, and another 181 articles were excluded after the assessment of full text. Finally, 35 studies [3–11, 15–38, 49, 50] met our inclusion criteria that were selected for the present meta-analysis.

### Study characteristics

These studies included a total of 8984 patients with a median age of 60.6 years and a median follow-up period of 26.8 months. Table 1 and Additional file 1: Table S2 provide the basic and summarized characteristics of the identified studies that met the inclusion criteria. In summary, all studies had a retrospective study design and were published between 2014 and 2018. 12 different kinds of epithelial tumors were included in these studies, and the median number of patients was 177. Colorectal cancer and lung cancer were the two main types of cancers. The main therapeutic strategies included chemotherapy, surgery and combined therapy. 28 studies were conducted in Asia, 5 in Europe and 2 in America and others. The association between pretreatment LMR and OS was investigated in all the included studies, among which 9 also investigated the association between pretreatment LMR and PFS as well. The median cut-off value for LMR was 3.23. Most of the included studies (32/35) used multivariate analysis method to adjust covariates when analyzing the prognostic value of LMR. According to the risk assessment scale, 3 studies had quality scores less than 7, the other 32 had a score more than 7 (Additional file 1: Table S3).

**Table 1:** Baseline characteristics of included studies (n = 35)

No.of Refs.	Authors (year)	Country	Inclusion period	Study design	Number of cases (F/M)	Median Age (years) (range)	Tumor type (stage)	Treatment	LMR cutoff value	Follow-up period (months)	End points	Analysis of hazard ratio	Adjusted variables	Quality score
3	Chen et al. (2015)	China	2011–2013	Retrospective	253 (104/149)	65.2	Non-Small Cell Lung Cancer (IIIB, IV)	Molecular targeted	3.29	24.02	PFS, OS	Multivariable	Distant metastasis, Malignant effusion, PS	8
17	Qi et al. (2015)	China	2011–2013	Retrospective	211 (134/77)	61.2	Pancreatic cancer (III, IV)	Chemo	3.3	NR	OS	Multivariable	Tumor stage, CA19-9	7
18	Song et al. (2015)	Korea	2006–2013	Retrospective	177 (83/94)	52 (25–81)	Colorectal cancer (IV)	Herbal medication, Acupuncture	3.4	3.1 (0.1–33.3)	OS	Multivariable	mGPS, CA19-9, Aspartate aminotransferase, Korean medicine treatment duration	7
19	Jiang et al. (2015)	China	2003–2009	Retrospective	672 (546/126)	46 (13–79)	Nasopharyngeal carcinoma (IV)	Chemo, Radio	2.475	NR	OS	Multivariable	N stage, No. of metastatic lesions, Liver metastasis	7
4	Lin et al. (2014)	China	2006–2010	Retrospective	256 (179/77)	53.6 (35–69)	Nasopharyngeal carcinoma (IV)	Chemo	5.07	22.6 (5.1–42.3)	OS	Multivariable	Age, ECOG performance status, Liver metastasis, No. of metastatic sites	8
15	Facciorusso et al. (2016)	Italy	2003–2012	Retrospective	127 (88/39)	66 (38–88)	Colorectal cancer (IV)	RF ablation	3.96	63 (54–71)	OS	Multivariable	Neutrophil-to-lymphocyte ratio, CEA, No. of nodules, Max diameter	8
5	Minami et al. (2017)	Japan	2007–2017	Retrospective	152 (57/95)	70.3	Non-Small Cell Lung Cancer (III, IV)	Molecular targeted	5.09	NR	PFS, OS	Multivariable	Distant metastasis, ECOG PS, BMI, EGFR-TKI line, Ccr, Sodium, LDH, CRP	6
20	Zhu et al. (2017)	China	2008–2015	Retrospective	672	55 (30–70)	Epithelial ovarian cancer (III, IV)	Chemo	3.45	38 (5–103)	PFS, OS	Multivariable	FIGO stage, CA-125, Chemosensitivity, Residual tumor	8
21	Li et al. (2016)	China	2003–2004	Retrospective	424	47 (18–74)	Cervical carcinoma (II-IV)	Radio, Chemo	5.28	73	PFS, OS	Multivariable	HPV DNA status, FIGO classification, pathological type, Lymph node status classification	8
22	Fukuda et al. (2018)	Japan	1986–2015	Retrospective	152 (109/43)	64	Renal cell carcinoma (IV)	Surgery	3.23	14	OS	Univariate	–	8
23	Li et al. (2017)	China	2008–2014	Retrospective	122	NR	Hepatocellular carcinoma (III)	Surgery	3	NR	OS	Multivariable	Barcelona clinic liver cancer, Tumor size, Tumor stage, Pathological differentiation	7
24	Peng et al. (2017)	China	2000–2012	Retrospective	150 (97/53)	58 (20–82)	Colorectal cancer (IV)	Chemo, Surgery	2.82	36 (2–126)	OS	Multivariable	Age, Lymph node metastases, Timing of metastasis, No. of metastatic tumors, Largest tumor size, Tumor distribution	8
14	Yang et al. (2017)	China	2009–2015	Retrospective	95 (58/37)	56 (27–86)	Colorectal cancer (IV)	Chemo, Molecular targeted	4	40 (12–72)	PFS, OS	Univariate	–	8
6	Lin et al. (2014)	China	2004–2012	Retrospective	370 (213/157)	63.6 (36–72)	Non-Small Cell Lung Cancer (IIIB, IV)	Chemo	4.56	NR	PFS, OS	Multivariable	Histology	6
25	Neal et al. (2015)	UK	2006–2010	Retrospective	302 (192/110)	64.8 (26–85)	Colorectal cancer (IV)	Surgery	2.35	29.7 (4–96)	OS	Univariate	–	8
26	Wu et al. (2016)	China	2009–2013	Retrospective	221	NR	Rectal cancer (III)	Surgery	5.13	NR	OS	Multivariable	Age, CEA, Tumor location, Differentiation, Vascular invasion, Perineural invasion	7
7	Lin et al. (2016)	China	2005–2013	Retrospective	488 (266/222)	54 (37–72)	Colorectal cancer (IV)	Chemo	3.11	23.5 (4.3–32.8)	PFS, OS	Multivariable	Gender, ECOG performance status, No. of metastatic sites, Tumor differentiation	8
27	Gu et al. (2016)	China	2006–2013	Retrospective	161 (128/33)	56 (17–83)	Renal cell carcinoma (IV)	Surgery	3.23	NR	OS	Multivariable	T stage, Fuhrman grade, Histology, Tumor necrosis, Targeted therapy, Hemoglobin c	6
8	Xiong et al. (2017)	China	2012–2015	Retrospective	78 (36/42)	59 (28–82)	Lung adenocarcinoma (IIIB, IV)	Chemo	4.3	15.3 (1.7–37.6)	PFS, OS	Multivariable	Gender, Smoking status, Clinical response, No. of metastasis organs	8
49	Yu et al. (2017)	China	2010–2013	Retrospective	139 (83/56)	NR	Pancreatic cancer (III, IV)	Chemo	3.19	78	OS	Multivariable	Stage, CA19-9, LDH	8
28	Chang et al. (2017)	China	2010–2014	Retrospective	490 (238/252)	63.8	Non-Small Cell Lung Cancer (IV)	Molecular targeted, Chemo	3.1	NR	OS	Multivariable	BMI, Sex, Diabetes mellitus, PS, EGFR mutation, Tumor type, De novo liver metastases	7
50	Liu et al. (2017)	China	2012–2013	Retrospective	162 (127/35)	63 (38–70)	Esophageal cancer (II, III)	Chemo, Radio	4.02	23.3 (8–43.7)	PFS, OS	Multivariable	cT status, Tumor stage, Tumor response	8
29	Stotz et al. (2014)	Austria	1996–2011	Retrospective	372 (217/155)	64 (27–95)	Colon cancer (II, III)	Surgery	2.83	68 (1–190)	OS	Multivariable	Tumor invasion depth, Lymph node involvement, Tumor stage	8
10	Neofytou et al. (2015)	UK	2005–2012	Retrospective	140 (88/52)	NR	Colorectal cancer (IV)	Surgery, Chemo	3	33 (1–103)	OS	Multivariable	Distribution of lesions, Lymph node-positive primary tumor, Adjuvant chemotherapy	8
30	Kozak et al. (2017)	USA	2005–2009	Retrospective	53	NR	Colorectal Cancer (III)	Surgery	2.6	NR	OS	Multivariable	Age, Overall Stage, Total lymph nodes	7
31	Shibutani et al. (2018)	Japan	2008–2016	Retrospective	160 (86/74)	65 (18–89)	Colorectal cancer (IV)	Chemo, Molecular targeted	2.96	21.8 (1.2–94.0)	OS	Multivariable	Sex, PS, Location of primary tumor, RAS status	7
9	Marin Hernández et al. (2018)	Spain	2003–2016	Retrospective	150	49.8 (28–77)	Breast cancer (II, III)	Chemo	5.46	24 (1–144)	OS	Multivariable	Pretreatment size, Neutrophil-to-lymphocyte ratio, Neutrophils	7
32	Xue et al. (2017)	China	2009–2015	Retrospective	153 (102/51)	60 (34–86)	Pancreatic cancer (III, IV)	Chemo	2.8	8.8 (0.5–75.5)	OS	Multivariable	ECOG PS, TNM stage, CA 19-9	7
33	Zhou et al. (2014)	China	2006–2008	Retrospective	426 (304/122)	NR	Gastric cancer (II, III)	Surgery, Chemo	4.32	39.58 (2.63–85.63)	OS	Multivariable	Size, Vascular/nerve infiltration, Resection margin, TNM stage, Adjuvant chemotherapy	8
33	Chan et al. (2017)	Australia	1998–2012	Retrospective	740 (370/370)	NR	Colorectal Cancer (III)	Surgery, Chemo, Radio	2.38	NR	OS	Multivariable	Age, T Stage, N stage, Grade, MMR-BRAF status	7
35	Oh et al. (2017)	Korea	200–2011	Retrospective	261 (143/118)	65.0 (31–86)	Colorectal cancer (II)	Surgery	3.7	78.0 (3–119)	OS	Multivariable	Age, Lymphatic invasion, Venous invasion, Perineural invasion, Preoperative CEA, Adjuvant chemotherapy	8
37	Kano et al. (2017)	Japan	2003–2012	Retrospective	222	NR	Head and neck cancer (III, IV)	Radio, Chemo	3.22	NR	OS	Multivariable	Age, Sex, Primary location, Chemotherapy	7
36	Cong et al. (2016)	China	2007–2011	Retrospective	188 (147/41)	77 (75–88)	Gastric cancer (II, III)	Surgery	4.34	21.8 (1.3–92.9)	OS	Multivariable	Gender, CEA, CA19-9, Tumor site, Tumor size, TNM, Lymph node metastasis	7
38	Li et al. (2016)	China	2012–2014	Retrospective	68	NR	Pancreatic adenocarcinoma (III)	Surgery	2.86	NR	OS	Multivariable	ASA score, T stage, Lymph node status, TNM stage, Pathological differentiation	7
11	Qi et al. (2016)	China	2009–2010	Retrospective	177 (108/69)	58.8	Chemo	3		NR	OS	Multivariable	Cancer stage, CA 19-9	7

*ASA* American Society of Anesthesiologists, *BMI* body mass index, *CA-125* cancer antigen 125, *CA19-9* carbohydrate antigen 19-9, *Ccr* creatinine clearance, *CEA* carcinoembryonic antigen, *Chemo* chemotherapy, *CRP* C-reactive protein, *DNA* deoxyribonucleic acid, *ECOG* Eastern Cooperative Oncology Group, *EGFR* epidermal growth factor receptor, *EGFR-TKI* epidermal growth factor receptor-tyrosine kinase inhibitors, *FIGO* International Federation of Gynecology and Obstetrics, *F/M* female/male, *HPV* human papillomavirus, *LDH* lactate dehydrogenase, *LMR* lymphocyte-to-monocyte ratio, *mGPS* modified Glasgow prognostic score, *No.* number, *NR* not reported, *OS* overall survival, *PFS* progression-free survival, *PS* performance status, *Radio* radiotherapy, *Ref.* reference; *RF* radiofrequency, *TNM* tumor node metastasis

### Primary outcome: overall survival

**Fig. 2:**
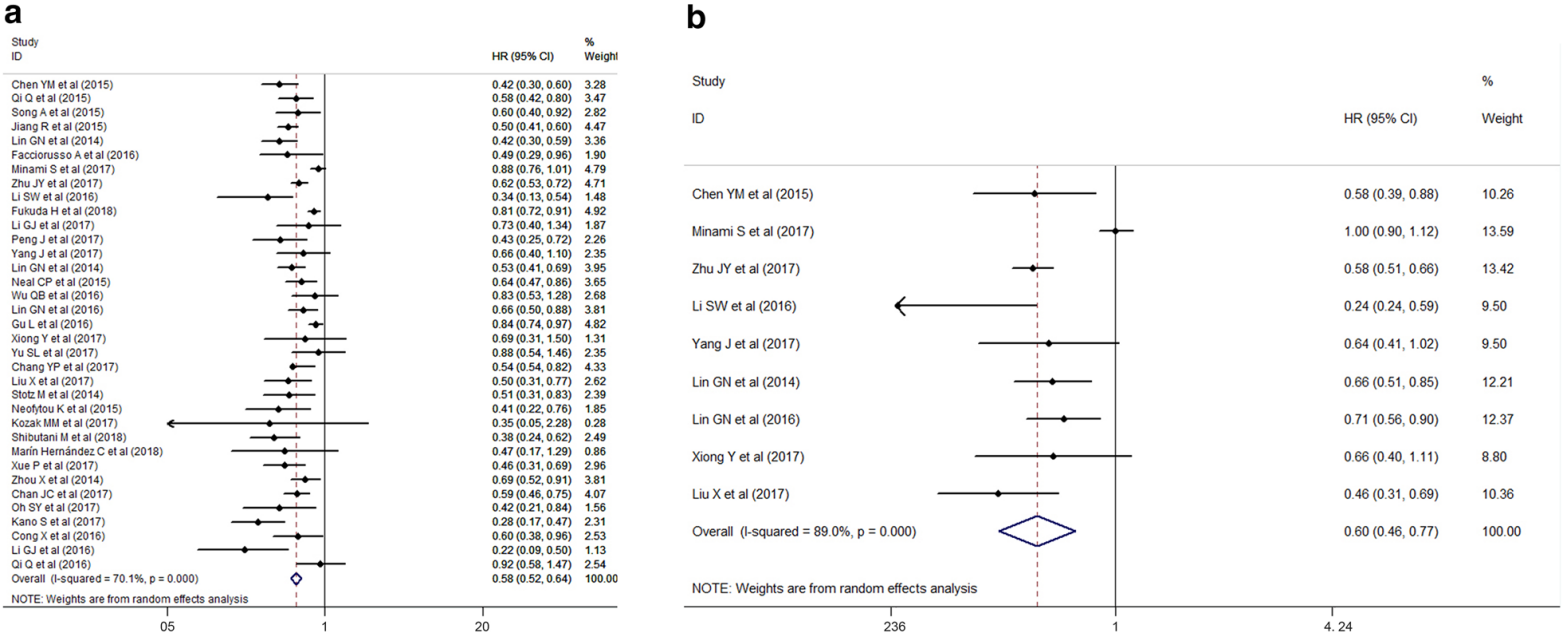
Meta-analysis of the associations between pretreatment blood LMR and **a** overall survival; **b** progression-free survival

35 studies with 8984 individuals were included in the analysis of pretreatment LMR and OS. Figure 2a indicates that a higher pretreatment LMR was associated with improved OS (HR = 0.578, 95% CI 0.522–0.641, *P* < 0.001). Given that the test for heterogeneity was significant (*Q *=113.56, *P* < 0.001, *I*^*2*^ = 70.1%), a random-effect model was used. Subgroup analyses were applied to explore potential sources of heterogeneity among several related clinical features for OS (Table 2). The pooled HRs of most subgroups were markedly changed in subgroup analyses. The subgroup analysis by tumor types showed a higher pretreatment LMR was significantly associated with better OS in colorectal cancer (n = 13, HR = 0.579, 95% CI 0.516–0.650, *I*^*2*^ = 0%), lung cancer (n = 5, HR = 0.594, 95% CI 0.435–0.811, *I*^*2*^ = 85.5%), pancreatic cancer (n = 5, HR = 0.588, 95% CI 0.407–0.851, *I*^*2*^ = 67.9%), gastric cancer (n = 2, HR = 0.664, 95% CI 0.523–0.843, *I*^*2*^ = 0%), nasopharyngeal carcinoma (n = 2, HR = 0.479, 95% CI 0.406–0.566, *I*^*2*^ = 0%), renal cancer (n = 2, HR = 0.827, 95% CI 0.755–0.906, *I*^*2*^ = 0%), cervical carcinoma (n = 1, HR = 0.337, 95% CI 0.164–0.691), ovarian cancer (n = 1, HR = 0.615, 95% CI 0.527–0.718), esophageal cancer (n = 1, HR = 0.495, 95% CI 0.315–0.778) and head and neck cancer (n = 1, HR = 0.28, 95% CI 0.168–0.466), but not breast cancer (n = 1, HR = 0.47, 95% CI 0.171–1.295, *P *= 0.144) and hepatocellular carcinoma (n = 1, HR = 0.73, 95% CI 0.399–1.336, *P * = 0.308). To be noted, the subgroup analysis by different therapeutic strategies indicated that a higher pretreatment LMR was associated with improved OS in chemotherapy (n = 10, HR = 0.592, 95% CI 0.518–0.676, *P *< 0.001), surgery (n = 10, HR = 0.683, 95% CI 0.579–0.807, *P *< 0.001), combined therapy (n = 11, HR = 0.507, 95% CI 0.442–0.582, *P *< 0.001) which consists of surgery and (neo)adjuvant therapy. The cut-off values of LMR in the studies ranged from 2.35 to 5.46. After stratifying the cut-off values of LMR into two subgroups, < 3.0 and ≥ 3.0, we noted that the level of statistical heterogeneity (< 3.0, *I*^*2*^ = 16%; ≥ 3.0, *I*^*2*^ = 69.8%) was reduced, while the pooled HRs were not significantly altered. The reduction in statistical heterogeneity was also realized after adjusting research region (Asia, *I*^*2*^ = 74.9%; Europe, *I*^*2*^ =  0%; America and others, *I*^*2*^ = 0%), number of cases (< 200, *I*^*2*^ = 63.2%; > 200, *I*^*2*^ = 41.3%), therapeutic strategies (Chemotherapy, *I*^*2*^ = 31.9%; Molecular targeted, *I*^*2*^ = 93%; Surgery, *I*^*2*^ = 54.3%; Combined therapy, *I*^*2*^ = 37.9%; others, *I*^*2*^ = 0%) and follow-up period (≤ 33, *I*^*2*^ = 67.8%; > 33, *I*^*2*^ = 15.6%; NR, *I*^*2*^ = 81.1%). Meanwhile, the subgroup analysis by publication year, initial inclusion period, median age, quality score and analysis of HR indicated that a high pretreatment LMR was consistently associated with superior OS.

**Table 2:** Subgroup Analyses of the Associations between LMR and overall survival

Variables	No. of studies	Test of association			Test of heterogeneity	
		HR	95% CI	*P* value	*I*^*2*^ (%)	*P* value
Total	35	0.578	0.522–0.641	< 0.001	70.10	< 0.001
Publication year						
≤ 2016	18	0.572	0.497–0.660	< 0.001	67.20	< 0.001
> 2016	17	0.583	0.499–0.681	< 0.001	72.60	< 0.001
Initial inclusion period						
≤ 2006	19	0.554	0.480–0.640	< 0.001	72.90	< 0.001
> 2006	16	0.604	0.516–0.707	< 0.001	68.20	< 0.001
Research region						
Asia	28	0.584	0.520–0.656	< 0.001	74.90	< 0.001
Europe	5	0.553	0.446–0.685	< 0.001	0.00	0.712
America and others	2	0.584	0.459–0.744	< 0.001	0.00	0.596
Number of cases						
< 200	19	0.632	0.547–0.730	< 0.001	63.20	< 0.001
> 200	16	0.549	0.497–0.606	< 0.001	41.30	0.043
Median age (years)						
≤ 60	13	0.585	0.496–0.691	< 0.001	69.20	< 0.001
> 60	13	0.575	0.488–0.679	< 0.001	75.70	< 0.001
NR	9	0.557	0.427–0.727	< 0.001	62.60	0.006
Tumor types						
Breast cancer	1	0.47	0.171–1.295	0.144	–	–
Cervical carcinoma	1	0.337	0.164–0.691	0.003	–	–
Colon cancer and rectal cancer	13	0.579	0.516–0.650	< 0.001	0.00	0.496
Ovarian cancer	1	0.615	0.527-0.718	< 0.001	–	–
Esophageal cancer	1	0.495	0.315– 0.778	0.002	–	–
Gastric cancer	2	0.664	0.523–0.843	0.001	0.00	0.622
Head and neck cancer	1	0.28	0.168–0.466	< 0.001	–	–
Hepatocellular carcinoma	1	0.73	0.399–1.336	0.308	–	–
Lung cancer	5	0.594	0.435–0.811	0.001	85.50	< 0.001
Nasopharyngeal carcinoma	2	0.479	0.406–0.566	< 0.001	0.00	0.379
Pancreatic cancer	5	0.588	0.407–0.851	0.005	67.90	0.014
Renal cancer	2	0.827	0.755–0.906	< 0.001	0.00	0.7
LMR cutoff						
< 3.0	9	0.508	0.444–0.582	< 0.001	16.00	0.3
≥ 3.0	26	0.612	0.546–0.686	< 0.001	69.80	< 0.001
Therapeutic strategies						
Chemotherapy	10	0.592	0.518–0.676	< 0.001	31.90	0.153
Molecular targeted	2	0.622	0.304–1.271	0.193	93.00	< 0.001
Surgery	10	0.683	0.579–0.807	< 0.001	54.30	0.02
Combined therapy	11	0.507	0.442–0.582	< 0.001	37.90	0.097
Others	2	0.563	0.400–0.794	0.001	0.00	0.577
Follow-up period (months)						
≤ 33	13	0.545	0.454–0.653	< 0.001	67.80	< 0.001
> 33	9	0.594	0.515–0.685	< 0.001	15.60	0.303
NR	13	0.606	0.504–0.729	< 0.001	81.10	< 0.001
Quality score						
< 7	3	0.753	0.592–0.958	0.021	83.20	0.003
≥ 7	32	0.558	0.504–0.619	< 0.001	58.30	< 0.001
Analysis of hazard ratio						
Multivariate	32	0.562	0.503–0.628	< 0.001	68.70	< 0.001
Univariate	3	0.755	0.643–0.887	0.001	23.30	0.272

*CI* confidence interval, *HR* hazard ratio, *No*. number, *LMR* lymphocyte-to-monocyte ratio

**Fig. 3:**
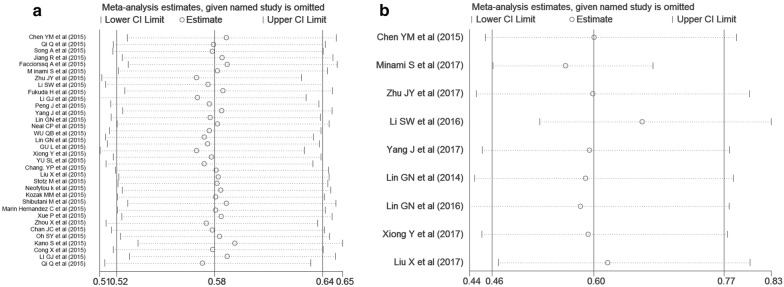
Sensitivity analysis for meta-analysis of the associations between pretreatment blood LMR and **a** overall survival; **b** progression-free survival

Sensitivity analysis on the stability of the OS subset indicated that omitting any single study did not significantly affect the pooled HRs (Fig. 3a). As shown in Additional file 2: Figure S1A, the asymmetrical funnel plot suggested that there could be publication bias. It was further confirmed with Egger’s test (Begg’s test, *P *= 0.334; Egger’s test, *P* < 0.001). The adjusted random effects pooled HRs of 0.578 (95% CI 0.522–0.641), obtained using the trim-and-fill method, which was consistent with our primary analysis (Additional file 1: Table S4). The funnel plot adjusted with trim-and-fill methods was shown in Additional file 2: Figure S1B.

### Secondary outcome: progression-free survival

Nine studies with 2694 individuals were included in the analysis of pretreatment LMR and PFS. Figure 2b demonstrates that a high pretreatment LMR was associated with longer PFS (HR = 0.598, 95% CI 0.465–0.768, *P* < 0.001). Since the test for heterogeneity was significant (*Q *= 72.92, *P* < 0.001, *I*^*2*^ = 89.0%), a random-effect model was used. Table 3 gives the results of subgroup analyses on potential sources of heterogeneity among several related clinical features of the included studies for PFS. The subgroup analysis by tumor types indicated that a higher pretreatment LMR was significantly associated with better PFS in colorectal cancer (n = 2, HR = 0.695, 95% CI 0.562–0.861, *I*^*2*^ = 0%), cervical carcinoma (n = 1, HR = 0.239, 95% CI 0.151–0.379), ovarian cancer (n = 1, HR = 0.581, 95% CI 0.508–0.664) and esophageal cancer (n = 1, HR = 0.461, 95% CI 0.31–0.685) but not lung cancer (n = 4, HR = 0.738, 95% CI 0.54–1.007, *I*^*2*^ = 80.00%, *P *= 0.056). A higher pretreatment LMR was proved to be associated with improved PFS in the subgroup analysis by different therapeutic strategies including chemotherapy (n = 4, HR = 0.62, 95% CI 0.558–0.688, *P *< 0.001) and combined therapy (n = 3, HR = 0.415, 95% CI 0.241–0.716, *P *= 0.002). The cut-off values of LMR ranged from 3.11 to 5.28 in different studies. The pooled HRs were not significantly altered by stratifying the cut-off values of LMR into 2 subgroups: ≤ 4.0 and > 4.0, which decreased the level of statistical heterogeneity (≤ 4.0, *I*^*2*^ =  0%; > 4.0, *I*^*2*^ = 92.2%) nonetheless. It was noted that the significant difference was altered in subgroup analysis by number of cases (< 200, *P* = 0.075), therapeutic strategies (molecular targeted, *P *= 0.384), follow-up period (NR, *P *= 0.356), quality score (< 7, *P *= 0.356) and analysis of hazard ratio (Univariate, *P *= 0.061). Sensitivity analysis further confirmed that omitting any single study did not significantly affect the pooled HRs, exhibiting good stability of PFS subset (Fig. 3b).

**Table 3:** Subgroup Analyses of the Associations between LMR and progression free survival

Variables	No. of studies	Test of association			Test of heterogeneity	
		HR	95% CI	*P* value	*I*^*2*^ (%)	*P* value
Total	9	0.598	0.465–0.768	< 0.001	89.00	< 0.001
Publication year						
≤ 2016	4	0.526	0.355–0.777	0.001	83.10	< 0.001
> 2016	5	0.66	0.470–0.881	0.006	89.60	< 0.001
Initial inclusion period						
≤ 2006	3	0.502	0.299–0.843	0.009	88.70	< 0.001
> 2006	6	0.648	0.47–0.882	0.006	89.60	< 0.001
Nationality						
China	8	0.561	0.466–0.675	< 0.001	64.40	0.006
Japan	1	1	0.896–1.116	1	–	–
Number of cases						
< 200	4	0.684	0.450–1.040	0.075	83.10	< 0.001
> 200	5	0.551	0.429–0.707	< 0.001	77.60	0.001
Median age (years)						
≤ 60	5	0.546	0.408–0.729	< 0.001	77.00	0.002
> 60	4	0.67	0.462–0.971	0.035	87.70	< 0.001
Tumor types						
Cervical carcinoma	1	0.239	0.151–0.379	< 0.001	–	–
Colon cancer and rectal cancer	2	0.695	0.562–0.861	0.001	0.00	0.713
Ovarian cancer	1	0.581	0.508–0.664	< 0.001	–	–
Esophageal cancer	1	0.461	0.31–0.685	< 0.001	–	–
Lung cancer	4	0.738	0.54–1.007	0.056	80.00	0.002
LMR cutoff						
≤ 4.0	4	0.609	0.546–0.680	< 0.001	0.00	0.546
> 4.0	5	0.56	0.351–0.892	0.015	92.20	< 0.001
Therapeutic strategies						
Chemotherapy	4	0.62	0.558–0.688	< 0.001	0.00	0.489
Molecular targeted	2	0.793	0.472–1.335	0.384	84.10	0.012
Combined therapy	3	0.415	0.241–0.716	0.002	78.40	0.01
Follow-up period (month)						
≤ 33	4	0.619	0.510–0.751	< 0.001	14.20	0.321
> 33	3	0.457	0.270–0.774	0.004	85.40	0.001
NR	2	0.826	0.55–1.239	0.356	88.50	0.003
Quality score						
< 7	2	0.826	0.550–1.239	0.356	88.50	0.003
≥ 7	7	0.542	0.435–0.674	< 0.001	67.80	0.005
Analysis of hazard ratio						
Multivariate	8	0.592	0.452–0.776	< 0.001	90.40	< 0.001
Univariate	1	0.644	0.406–1.021	0.061	–	–

*CI* confidence interval, *HR* hazard ratio, *No*. number; *LMR* lymphocyte-to-monocyte ratio

## Discussion

A low LMR was first reported to be a poor prognostic indicator in patients with hematologic malignancies [51]. In recent years, several meta-analysis were performed to analyze the relationship between LMR and clinical outcomes of non-hematologic solid tumors [51, 52]. Nishijima et al. first performed a meta-analysis to quantify the prognostic value of pretreatment LMR in non-hematologic solid tumors without incorporating any confounding variable at the patient level or quality of studies into their analysis [51]. Teng et al. carried out another study on the same theme, by using advanced statistical methods, while not making subgroup analysis on different therapeutic strategies [52]. Furthermore, given that solid cancers originate from either epithelium or mesenchyme, it is reasonable and necessary to further assess the prognostic value of LMR in advanced-stage epithelial cancers.

To our best knowledge, this is the first meta-analysis to evaluate the association between LMR and outcomes of advanced epithelial cancer patients including the search results from 4 available databases online. We included 35 studies comprising 8984 patients with advanced epithelial tumors and found that a high pretreatment LMR was associated with favorable OS (HR = 0.578, 95% CI 0.522–0.641, *P* < 0.001) and PFS (HR = 0.598, 95% CI 0.465–0.768, *P* < 0.001). Furthermore, subgroup analyses were based on publication year, types of cancers, cut-off value, median age, initial inclusion period, research region, treatment, follow-up period, quality score and analysis of hazard ratio. The association between pretreatment LMR and OS remained mostly constant in various subgroups. Notably, the pooled HRs as well as 95% CI were statistically significant in the subgroups of therapeutic strategies, except for molecular targeted therapy, which may be attributed to the limited number of studies. Therefore, the study revealed that pretreatment LMR might serve as a discriminative indicator for the prognoses of patients who undergo different therapeutic strategies.

The internal mechanisms of high pretreatment LMR associated with favorable outcomes of cancer patients remained unclear. The association may be explained through immune inflammation in the tumor microenvironment. It is well recognized that inflammation plays important roles in various cancers [2]. Tumor-infiltrating lymphocytes (TILs) and tumor-associated macrophages (TAMs) are common inflammatory cells in the tumor milieu that have been found to be prognostic factors [53–55]. TILs participate in cellular as well as humoral antitumor immune responses that contribute to tumor control. Furthermore, high numbers of TILs are associated with improved outcomes [56–59]. In addition, TILs are potential targets for cancer immunotherapy in several cancer types, including non-small-cell lung carcinoma, colorectal cancer, cutaneous T cell lymphoma and melanoma [57, 60–62]. Peripheral monocytes and myeloid progenitor cells differentiate into TAMs when entering tumors [14]. Shibutani et al. reported that the peripheral monocyte count is associated with the density of tumor-associated macrophages in the tumor microenvironment of colorectal cancer [12]. TAMs accelerate tumor progression and metastasis through production of growth factors and cytokines, which lead to angiogenesis and anti-immune responses [51, 52]. Studies indicated that high numbers of TAMs or pretreatment monocytes are associated with poor outcomes [14, 63–66]. Therefore, a high pretreatment LMR reflect a strong antitumor immunity in the tumor microenvironment and indicate latent therapeutic benefits for advanced-stage epithelial cancers.

Our study had several limitations. First, significant heterogeneity was observed among the included studies. Therefore, a random-effects model was used to adjust the heterogeneity in the analyses of OS and PFS. We also performed prespecified subgroup analyses to reduce the heterogeneity. Second, the number of studies included to assess the pretreatment LMR and outcomes undergoing different therapeutic strategies was limited, which could have led to the non-significant differences in subgroup analyses. Third, evidence of publication bias was inevitably observed, with fewer studies reporting negative results than would be expected. However, the random effects pooled HRs adjusted using the trim-and-fill methods did not shift the results in primary analysis. This suggests that our results are not biased by negative results. Moreover, HRs were available from only univariate analysis in 3 studies. These studies could lead to overestimation of the prognostic value of LMR, although sensitivity analysis indicated good stability of our results. Finally, the number of studies in the analysis of pretreatment LMR and PFS was small and the heterogeneity was also significant which may have biased our analysis.

Despite the above limitations, our meta-analysis supports the values of LMR as a promising independent predictor of survival in advanced epithelial cancer patients. Since LMR can be obtained from routine blood tests, intermediate assessments about changes in LMR during therapy are simply available. Therefore LMR could be used to improve clinical decision-making regarding treatment in advanced epithelial cancers.

## Conclusion

Here, we searched online databases for relevant studies, and enrolled 35 studies with a total of 8984 patients for meta-analysis, drawing a conclusion that a high pretreatment LMR is associated with favorable survival with advanced-stage epithelial cancers undergoing different therapeutic strategies. A prospective trial is needed to identify LMR as a simple and readily available prognostic biomarker in clinical practice.

## Additional files

**Additional file 1.** Additional tables.

**Additional file 2: Figure S1.** Funnel plot for meta-analysis of the association between pretreatment blood LMR and (A) overall survival, (B) overall survival adjusted with trim-and-fill methods.
