# Supplementary material for: Prognostic impact of pretreatment lymphocyte-to-monocyte ratio in advanced epithelial cancers: a meta-analysis
Source: Cancer Cell Int. 2018 Dec 6;18:201. doi: 10.1186/s12935-018-0698-5 (PMC6282251; doi:10.1186/s12935-018-0698-5)
Supplement: Supplementary file 1 — Additional file 1. Additional tables. [file 12935_2018_698_MOESM1_ESM.docx]

**Additional Table S1. Search Strategies**

Search included: PUBMED,EMBASE databases, Web of science and Cochrane Library Search strategy：Search date was up to July 2018

**1) Pubmed search strategy**

| 1. "Neoplasms"[Mesh] |
| --- |
| 1. neoplas* [Title/Abstract] |
| 1. tumor* [Title/Abstract] |
| 1. tumour*[Title/Abstract] |
| 1. cancer*[Title/Abstract] |
| 1. carcinoma* [Title/Abstract] |
| 1. Neoplasm*, Malignant[Title/Abstract] |
| 1. Malignant neoplasm*[Title/Abstract] |
| 1. malignan*[Title/Abstract] |
| 1. 1 OR 2 OR 3 OR 4 OR 5 OR 6 OR 7 OR 8 OR 9 |
| 1. metasta*[Title/Abstract] |
| 1. advanced*[Title/Abstract] |
| 1. 11 OR 12 |
| 1. "Lymphocytes" [Mesh] |
| 1. Lymphocyte*[Title/Abstract] |
| 1. Lymphoid Cell*[Title/Abstract] |
| 1. Cell*, Lymphoid[Title/Abstract] |
| 1. 14 OR 15 OR 16 OR 17 |
| 1. "Monocytes"[Mesh] |
| 1. Monocyte*[Title/Abstract] |
| 1. 19 OR 20 |
| 1. 18 AND 21 |
| 1. L/M ratio[Title/Abstract] |
| 1. lymphocyte to monocyte ratio[Title/Abstract] |
| 1. lymphocyte monocyte ratio[Title/Abstract] |
| 1. 22 OR 23 OR 24 OR 25 |
| 1. "Survival"[Mesh] |
| 1. survival[Title/Abstract] |
| 1. "Mortality"[Mesh] |
| 1. mortality[Title/Abstract] |
| 1. "Prognosis"[Mesh] |
| 1. prognos*[Title/Abstract] |
| 1. outcome*[Title/Abstract] |
| 1. recurren*[Title/Abstract] |
| 1. predict*[Title/Abstract] |
| 1. 27 OR 28 OR 29 OR30 OR 31 OR 32 OR 33 OR 34 OR 35 |
| 1. 10 AND 13 AND 26 AND 36 |

**2) Embase search strategy**

| 1. 'neoplasm'/exp |
| --- |
| 1. 'neoplas*':ab,ti |
| 1. 'tumor*':ab,ti |
| 1. 'tumour*':ab,ti |
| 1. 'cancer*':ab,ti |
| 1. 'carcinoma*':ab,ti |
| 1. 'malignan*':ab,ti |
| 1. 'malignant neoplasm*':ab,ti |
| 1. 'neoplasm*, malignant':ab,ti |
| 1. 1 OR 2 OR 3 OR 4 OR 5 OR 6 OR 7 OR 8 OR 9 |
| 1. 'metasta*':ab,ti |
| 1. 'advanced*':ab,ti |
| 1. 11 OR 12 |
| 1. 'lymphocyte'/exp |
| 1. 'lymphocyte*':ab,ti |
| 1. 'lymphoid cell*':ab,ti |
| 1. 'cell*, lymphoid':ab,ti |
| 1. 14 OR 15 OR 16 OR 17 |
| 1. 'monocyte'/exp |
| 1. 'monocyte*':ab,ti |
| 1. 19 OR 20 |
| 1. 18 AND 21 |
| 1. 'lymphocyte to monocyte ratio'/exp |
| 1. 'lymphocyte to monocyte ratio':ab,ti |
| 1. 'lymphocyte monocyte ratio':ab,ti |
| 1. 22 OR 23 OR 24 OR 25 |
| 1. 'survival'/exp |
| 1. 'survival':ab,ti |
| 1. 'mortality'/exp |
| 1. 'mortality':ab,ti |
| 1. 'prognosis'/exp |
| 1. 'prognos*':ab,ti |
| 1. 'outcome*':ab,ti |
| 1. ''recurren*':ab,ti |
| 1. 'predict*':ab,ti |
| 1. 27 OR 28 OR 29 OR 30 OR 31 OR 32 OR 33 OR 34 OR 35 |
| 1. 10 AND 13 AND 26 AND 36 |

**3) Web of science search strategy**

| 1. neoplas* Subject search |
| --- |
| 2.tumor* Subject search |
| 3. tumour* Subject search |
| 4. cancer* Subject search |
| 5. carcinoma* Subject search |
| 6. malignan* Subject search |
| - 1. OR 2 OR 3 OR 4 OR 5 OR 6 |
| 8.metasta* Subject search |
| 9.advanced* Subject search |
| - 1. OR 9 |
| 11. Lymphocyte* Subject search |
| 12. Lymphoid Cell* Subject search |
| 13.11 OR 12 |
| 14. Monocyte* Subject search |
| - 1. AND 14 |
| 16.L/M ratio* Subject search |
| 17.lymphocyte to monocyte ratio* Subject search |
| 18.lymphocyte monocyte ratio* Subject search |
| - 1. OR 16 OR 17 OR 18 |
| 20.Survival* Subject search |
| 21.Mortality* Subject search |
| 22.prognos* Subject search |
| 23.outcome* Subject search |
| 24.recurren* Subject search |
| 25. predict* Subject search |
| 26. 20 OR 21 OR 22 OR 23 OR 24 OR 25 |
| 27.7 AND 10 AND 19 AND 26 |

**4) Cochrane Library search strategy**

| 1. 'neoplasm' Mesh |
| --- |
| 1. 'neoplas*': Title,Abstract,Keywords |
| 1. 'tumor*': Title,Abstract,Keywords |
| 1. 'tumour*': Title,Abstract,Keywords |
| 1. 'cancer*': Title,Abstract,Keywords |
| 1. 'carcinoma*': Title,Abstract,Keywords |
| 1. 'malignan*': Title,Abstract,Keywords |
| 1. 1 OR 2 OR 3 OR 4 OR 5 OR 6 OR 7 |
| 1. 'metasta*': Title,Abstract,Keywords |
| 1. 'advanced*': Title,Abstract,Keywords |
| 1. 9 OR 10 |
| 1. 'lymphocytes' Mesh |
| 1. 'lymphocyte*': Title,Abstract,Keywords |
| 1. 'lymphoid cell*': Title,Abstract,Keywords |
| 1. 12 OR 13 OR 14 |
| 1. 'monocytes' Mesh |
| 1. 'monocyte*': Title,Abstract,Keywords |
| 1. 16 OR 17 |
| 1. 15 AND 18 |
| 1. 'lymphocyte to monocyte ratio' :Title,Abstract,Keywords |
| 1. 'lymphocyte monocyte ratio': Title,Abstract,Keywords |
| 1. 19 OR 20 OR 21 |
| 1. 'survival' Mesh |
| 1. 'survival': Title,Abstract,Keywords |
| 1. 'mortality' Mesh |
| 1. 'mortality': Title,Abstract,Keywords |
| 1. 'prognosis' Mesh |
| 1. 'prognos*': Title,Abstract,Keywords |
| 1. 'outcome*': Title,Abstract,Keywords |
| 1. ''recurren*': Title,Abstract,Keywords |
| 1. 'predict*': Title,Abstract,Keywords |
| 1. 23 OR 24 OR 25 OR 26 OR 27 OR 28 OR 29 OR 30 OR 31 |
| 1. 8 AND 11 AND 22 AND 32 |

| **Additional Table S2. The main characteristic of each study** | | | |
| --- | --- | --- | --- |
| **Characteristics** | **Number of studies (%)** | **Patients (%)** | **Sequence number of references in Table S1** |
| **Total** | 35(100%) | 8984(100%) | 1-35 |
| **Publication type, No. (%)** |  |  |  |
| Full text | 35(100%) | 8984(100%) | 1-35 |
| **Year of publication, No. (%)** |  |  |  |
| 2014 | 4(11.43%) | 1424(15.85%) | 5,14,23,29 |
| 2015 | 6(17.14%) | 1755(19.53%) | 1-4,15,24 |
| 2016 | 8(22.86%) | 1854(20.64%) | 6,9,16-18,33-35 |
| 2017 | 14(40%) | 3489(38.84%) | 7,8,11-13,19-22,25,28,30-32 |
| 2018 | 3(8.57%) | 462(5.14%) | 10,26,27 |
| **Initial inclusion period, No. (%)** |  |  |  |
| ≤2006 | 19(54.29%) | 5643(62.81%) | 3-6,9,10,12,14,15,17,18,23-25,27,29-32 |
| >2006 | 16(45.71%) | 3341(37.19%) | 1,2,7,8,11,13,16,19-22,26,28,33-35 |
| **Research region, No. (%)** |  |  |  |
| Asia | 28(80%) | 7100(79.03%) | 1-5,7-14,16-22,26,28,29,31-35 |
| Europe | 5(14.29%) | 1091(12.14%) | 6,15,23,24,27 |
| America and others | 2(5.71%) | 793(8.83%) | 25,30 |
| **Number of cases, No. (%)** |  |  |  |
| <200 | 19(54.29%) | 2604(28.98%) | 3,6,7,10-13,18-20,22,24-28,33-35 |
| >200 | 16(45.71%) | 6380(71.02%) | 1,2,4,5,8,9,14-17,21,23,29-32 |
| **Median age (years), No. (%)** |  |  |  |
| ≤60 | 13(37.14%) | 3653(40.66%) | 3-5,8,9,12,13,17-19,27,28,35 |
| >60 | 13(37.14%) | 3200(35.62%) | 1,2,6,7,10,14,15,21-23,26,31,33 |
| NR | 9(25.71%) | 2131(23.72%) | 11,16,20,24,25,29,30,32,34 |
| **Study design, No. (%)** |  |  |  |
| retrospective study | 35(100%) | 8984(100%) | 1-35 |
| **Tumor types, No. (%)** |  |  |  |
| breast cancer | 1(2.86%) | 150(1.67%) | 27 |
| cervical carcinoma | 1(2.86%) | 424(4.72%) | 9 |
| colon cancer and rectal cancer | 13(37.14%) | 3286(36.58%) | 3,6,12,13,15-17,23-26,30,31 |
| ovarian cancer | 1(2.86%) | 672(7.48%) | 8 |
| esophageal cancer | 1(2.86%) | 162(1.80%) | 22 |
| gastric cancer | 2(5.71%) | 614(6.84%) | 29,33 |
| head and neck cancer | 1(2.86%) | 222(2.47%) | 32 |
| hepatocellular carcinoma | 1(2.86%) | 122(1.36%) | 11 |
| lung cancer | 5(14.29%) | 1343(14.95%) | 1,7,14,19,21 |
| nasopharyngeal carcinoma | 2(5.71%) | 928(10.33%) | 4,5 |
| pancreatic cancer | 5(14.29%) | 748(8.33%) | 2,20,28,34,35 |
| renal cancer | 2(5.71%) | 313(3.48%) | 10,18 |
| **LMR cut-off, No. (%)** |  |  |  |
| 2.0 to <3.0 | 9(25.71%) | 2670(29.72%) | 4,12,15,23,25,26,28,30,34 |
| 3.0 to <4.0 | 15(42.86%) | 3792(42.21%) | 1-3,6,8,10,11,17,18,20,21,24,31,32,35 |
| ≥4.0 | 11(31.43%) | 2522(28.07%) | 5,7,9,13,14,16,19,22,27,29,33 |
| **Therapeutic strategies** |  |  |  |
| Chemotherapy | 10(28.57%) | 2694(29.99%) | 2,5,8,14,17,19,20,27,28,35 |
| Molecular targeted | 2(5.71%) | 405(4.51%) | 1,7 |
| Surgery | 10(28.57%) | 1900(21.15%) | 10,11,15,16,18,23,25,31,33,34 |
| Combined therapy | 11(31.43%) | 3681(40.97%) | 4,9,12,13,21,22,24,26,29,30,32 |
| Others | 2(5.71%) | 304(3.38%) | 3,6 |
| **Endpoints, No. (%)** |  |  |  |
| OS | 35(100%) | 8984(100%) | 1-35 |
| PFS | 9(25.71%) | 2694(29.99%) | 1,7-9,13,14,17,19,22 |
| **Follow-up period, No. (%)** |  |  |  |
| ≤33 | 13(37.14%) | 2659(29.60%) | 1,3,5,10,15,17,19,22,24,26-28,33 |
| >33 | 9(25.71%) | 2666(29.67%) | 6,8,9,12,13,20,23,29,31 |
| NR | 13(37.14%) | 3659(40.73%) | 2,4,7,11,14,16,18,21,25,30,32,34,35 |
| **Quality score, No. (%)** |  |  |  |
| 6 | 3(8.57%) | 683(7.60%) | 7,14,18 |
| 7 | 15(42.86%) | 3804(42.34%) | 2-4,11,16,21,25-28,30,32-35 |
| 8 | 17(48.57%) | 4497(50.06%) | 1,5,6,8-10,12,13,15,17,19,20,22-24,29,31 |
| **Analysis of hazard ratio, No. (%)** |  |  |  |
| Multivariate | 32(91.43%) | 8435(93.89%) | 1-9,11,12,14,16-35 |
| Univariate | 3(8.57%) | 549(6.11%) | 10,13,15 |
| CI: confidence interval; HR: hazard ratio; No. Number; LMR: lymphocyte-to-monocyte ratio | | | |

| **Additional Table S3. Quality assessment of included studies (n=35)** | | | | | | | | | | |
| --- | --- | --- | --- | --- | --- | --- | --- | --- | --- | --- |
| **No.** | **Author** | **Year of publication** | **Representativeness of population** | **Non exposed cohort** | **Ascertainment of exposure** | **Outcome not present at start of study** | **Appropriate confounding measurement and account** | **Sufficient measurement of outcomes** | **Completeness of follow-up** | **Quality score** |
| 1 | Chen YM et al | 2015 | 0 | 1 | 1 | 1 | 2 | 1 | 2 | 8 |
| 2 | Qi Q et al | 2015 | 0 | 1 | 1 | 1 | 2 | 1 | 1 | 7 |
| 3 | Song A et al | 2015 | 0 | 1 | 1 | 1 | 2 | 1 | 1 | 7 |
| 4 | Jiang R et al | 2015 | 0 | 1 | 1 | 1 | 2 | 1 | 1 | 7 |
| 5 | Lin GN et al | 2014 | 0 | 1 | 1 | 1 | 2 | 1 | 2 | 8 |
| 6 | Facciorusso A et al | 2016 | 0 | 1 | 1 | 1 | 2 | 1 | 2 | 8 |
| 7 | Minami S et al | 2017 | 0 | 1 | 1 | 1 | 2 | 1 | 0 | 6 |
| 8 | Zhu JY et al | 2017 | 0 | 1 | 1 | 1 | 2 | 1 | 2 | 8 |
| 9 | Li SW et al | 2016 | 0 | 1 | 1 | 1 | 2 | 1 | 2 | 8 |
| 10 | Fukuda H et al | 2018 | 0 | 1 | 1 | 1 | 2 | 1 | 2 | 8 |
| 11 | Li GJ et al | 2017 | 0 | 1 | 1 | 1 | 2 | 1 | 1 | 7 |
| 12 | Peng J et al | 2017 | 0 | 1 | 1 | 1 | 2 | 1 | 2 | 8 |
| 13 | Yang J et al | 2017 | 0 | 1 | 1 | 1 | 2 | 1 | 2 | 8 |
| 14 | Lin GN et al | 2014 | 0 | 1 | 1 | 1 | 2 | 1 | 0 | 6 |
| 15 | Neal CP et al | 2015 | 0 | 1 | 1 | 1 | 2 | 1 | 2 | 8 |
| 16 | Wu QB et al | 2016 | 0 | 1 | 1 | 1 | 2 | 1 | 1 | 7 |
| 17 | Lin GN et al | 2016 | 0 | 1 | 1 | 1 | 2 | 1 | 2 | 8 |
| 18 | Gu L et al | 2017 | 0 | 1 | 1 | 1 | 2 | 1 | 0 | 6 |
| 19 | Xiong Y et al | 2017 | 0 | 1 | 1 | 1 | 2 | 1 | 2 | 8 |
| 20 | Yu SL et al | 2017 | 0 | 1 | 1 | 1 | 2 | 1 | 2 | 8 |
| 21 | Chang YP et al | 2017 | 0 | 1 | 1 | 1 | 2 | 1 | 1 | 7 |
| 22 | Liu X et al | 2017 | 0 | 1 | 1 | 1 | 2 | 1 | 2 | 8 |
| 23 | Stotz M et al | 2014 | 0 | 1 | 1 | 1 | 2 | 1 | 2 | 8 |
| 24 | Neofytou K et al | 2015 | 0 | 1 | 1 | 1 | 2 | 1 | 2 | 8 |
| 25 | Kozak MM et al | 2015 | 0 | 1 | 1 | 1 | 2 | 1 | 1 | 7 |
| 26 | Shibutani M et al | 2018 | 0 | 1 | 1 | 1 | 2 | 1 | 1 | 7 |
| 27 | Marín Hernández C et al | 2017 | 0 | 1 | 1 | 1 | 2 | 1 | 1 | 7 |
| 28 | Xue P et al | 2017 | 0 | 1 | 1 | 1 | 2 | 1 | 1 | 7 |
| 29 | Zhou X et al | 2014 | 0 | 1 | 1 | 1 | 2 | 1 | 2 | 8 |
| 30 | Chan JC et al | 2017 | 0 | 1 | 1 | 1 | 2 | 1 | 1 | 7 |
| 31 | Oh SY et al | 2016 | 0 | 1 | 1 | 1 | 2 | 1 | 2 | 8 |
| 32 | Kano S et al | 2016 | 0 | 1 | 1 | 1 | 2 | 1 | 1 | 7 |
| 33 | Cong X et al | 2016 | 0 | 1 | 1 | 1 | 2 | 1 | 1 | 7 |
| 34 | Li GJ et al | 2016 | 0 | 1 | 1 | 1 | 2 | 1 | 1 | 7 |
| 35 | Qi Q et al | 2016 | 0 | 1 | 1 | 1 | 2 | 1 | 1 | 7 |

**Additional Table S4. The publication bias assessment with different tests for overall survival**

| **Publication bias** | **Begg’s *P* value** | **Egger’s *P* value** | **T&F(Fill)** |
| --- | --- | --- | --- |
| Overall survival | 0.334 | <0.001 | 0.578 (0.522-0.641) (0) |
| Abbreviations: Fill= number of studies added by trim and fill method; T&F= result of trimmed and filled analysis, using assumption of random effects. | | | |

| **Additional Table S5. Methods to determine the cut-off value of LMR (n=35)** | |  |  |
| --- | --- | --- | --- |
| No.of Ref. | Authors (year) | | Statistical Methods |
| 3 | Chen YM et al (2015) | | The cut-off value of LMR was determined by receiver operating characteristic (ROC) curve analysis |
| 17 | Qi Q et al (2015) | | The median level was used as the cut-off value for LMR |
| 18 | Song A et al (2015) | | The cut-off value of LMR was determined by receiver operating characteristic (ROC) curve analysis |
| 19 | Jiang R et al (2015) | | The cut-off value of LMR was determined by receiver operating characteristic (ROC) curve analysis |
| 4 | Lin GN et al (2014) | | The cut-off value of LMR was determined by receiver operating characteristic (ROC) curve analysis |
| 15 | Facciorusso A et al (2016) | | The LMR value with the highest log-rank statistical value was finally chosen as the optimal cut-off point |
| 5 | Minami S et al (2017) | | The cut-off value of LMR was determined by receiver operating characteristic (ROC) curve analysis |
| 20 | Zhu JY et al (2017) | | The cut-off value of LMR was determined by receiver operating characteristic (ROC) curve analysis |
| 21 | Li SW et al (2016) | | The cut-off value of LMR was determined by receiver operating characteristic (ROC) curve analysis |
| 22 | Fukuda H et al (2018) | | The cut-off value of LMR was determined by receiver operating characteristic (ROC) curve analysis |
| 23 | Li GJ et al (2017) | | The cut-off value of LMR was determined by receiver operating characteristic (ROC) curve analysis |
| 24 | Peng JH et al (2017) | | The cut-off value of LMR was determined by receiver operating characteristic (ROC) curve analysis |
| 14 | Yang J et al (2017) | | The median level was used as the cut-off value for LMR |
| 6 | Lin GN et al (2014) | | The cut-off value of LMR was determined by receiver operating characteristic (ROC) curve analysis |
| 25 | Neal CP et al (2015) | | The cut-off value of LMR was determined by receiver operating characteristic (ROC) curve analysis |
| 26 | Wu QB et al (2016) | | The median level was used as the cut-off value for LMR |
| 7 | Lin GN et al (2016) | | The cut-off value of LMR was determined by receiver operating characteristic (ROC) curve analysis |
| 27 | Gu LY et al (2016) | | The cut-off value of LMR was determined by receiver operating characteristic (ROC) curve analysis |
| 8 | Xiong YJ et al (2017) | | The cut-off value of LMR was determined by receiver operating characteristic (ROC) curve analysis |
| 49 | Yu SL et al (2017) | | The median level was used as the cut-off value for LMR |
| 28 | Chang YP et al (2017) | | The cut-off value of LMR was determined by receiver operating characteristic (ROC) curve analysis |
| 50 | Liu XM et al (2017) | | The median level was used as the cut-off value for LMR |
| 29 | Stotz M et al (2014) | | The cut-off value of LMR was determined by receiver operating characteristic (ROC) curve analysis |
| 10 | Neofytou K et al (2015) | | The cut-off value of LMR was determined by receiver operating characteristic (ROC) curve analysis |
| 30 | Kozak MM et al (2017) | | The median level was used as the cut-off value for LMR |
| 31 | Shibutani M et al (2018) | | The cut-off value of LMR was determined by receiver operating characteristic (ROC) curve analysis |
| 9 | Marin Hernández C et al (2018) | | The cut-off value of LMR was determined by receiver operating characteristic (ROC) curve analysis |
| 32 | Xue P et al (2017) | | The cut-off value of LMR was determined by receiver operating characteristic (ROC) curve analysis |
| 33 | Zhou X et al (2014) | | The cut-off value of LMR was determined by receiver operating characteristic (ROC) curve analysis |
| 33 | Chan JC et al (2017) | | MaxStat analysis was performed to find the optimal cut-off value for the LMR |
| 35 | Oh SY et al (2017) | | The cut-off value of LMR was determined by receiver operating characteristic (ROC) curve analysis |
| 37 | Kano S et al (2017) | | The cut-off value of LMR was determined by receiver operating characteristic (ROC) curve analysis |
| 36 | Cong X et al (2016) | | The cut-off value of LMR was determined by receiver operating characteristic (ROC) curve analysis |
| 38 | Li GJ et al (2016) | | The cut-off value of LMR was determined by receiver operating characteristic (ROC) curve analysis |
| 11 | Qi Q et al (2016) | | The cut-off value of LMR was determined by receiver operating characteristic (ROC) curve analysis |
| LMR: lymphocyte-to-monocyte ratio | | | |
