# Supplementary figures and images for: Prognostic impact of pretreatment lymphocyte-to-monocyte ratio in advanced epithelial cancers: a meta-analysis
Source: Cancer Cell Int. 2018 Dec 6;18:201. doi: 10.1186/s12935-018-0698-5 (PMC6282251; doi:10.1186/s12935-018-0698-5)

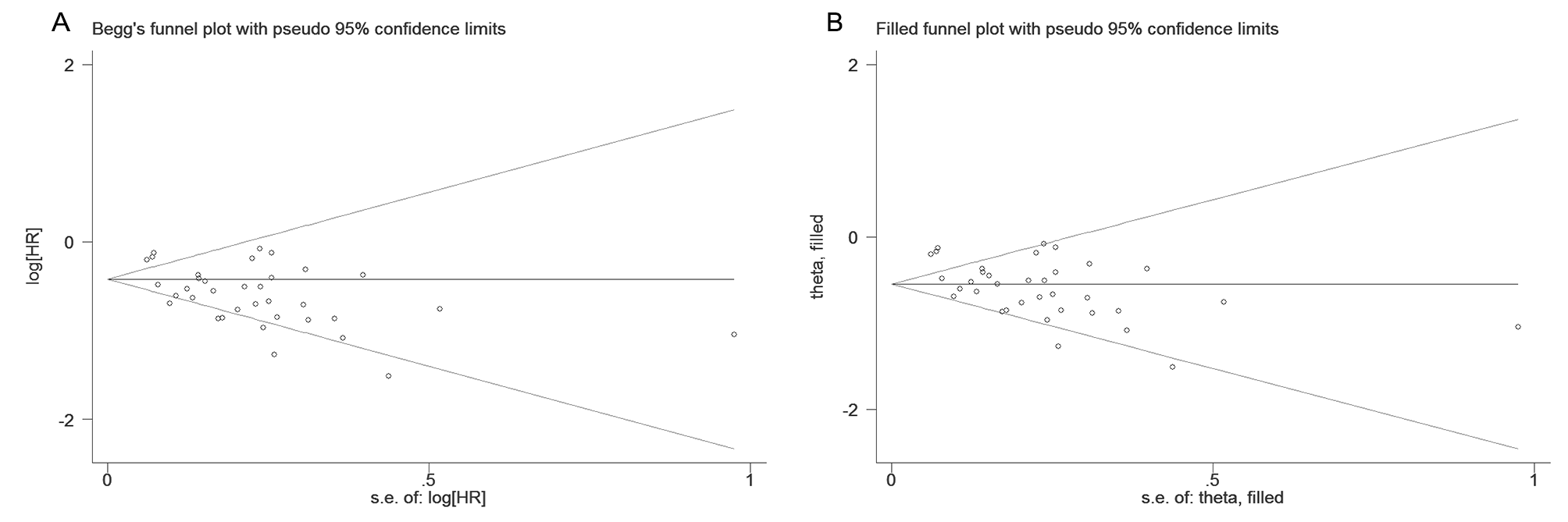

Supplement: Supplementary file 2 — Additional file 2: Figure S1. Funnel plot for meta-analysis of the association between pretreatment blood LMR and (A) overall survival, (B) overall survival adjusted with trim-and-fill methods. [file 12935_2018_698_MOESM2_ESM.tif]
